# Recent Advances in Chemical Modification of Peptide Nucleic Acids

**DOI:** 10.1155/2012/518162

**Published:** 2012-09-06

**Authors:** Eriks Rozners

**Affiliations:** Department of Chemistry, Binghamton University, State University of New York, 4400 Vestal Parkway East, Binghamton, NY 13902, USA

## Abstract

Peptide nucleic acid (PNA) has become an extremely powerful tool in chemistry and biology. Although PNA recognizes single-stranded nucleic acids with exceptionally high affinity and sequence selectivity, there is considerable ongoing effort to further improve properties of PNA for both fundamental science and practical applications. The present paper discusses selected recent studies that improve on cellular uptake and binding of PNA to double-stranded DNA and RNA. The focus is on chemical modifications of PNA's backbone and heterocyclic nucleobases. The paper selects representative recent studies and does not attempt to provide comprehensive coverage of the broad and vibrant field of PNA modification.

## 1. Introduction

**Figure 1:**
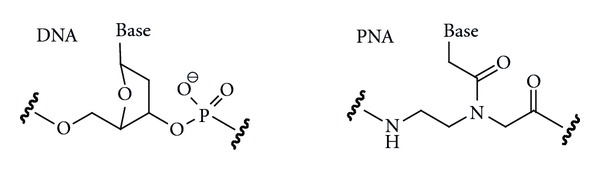
Structures of DNA and PNA repeating units.

Peptide nucleic acid (PNA) is a DNA analogue that has the entire sugar-phosphodiester backbone replaced by a pseudopeptide linkage built of 2-aminoethylglycine residues (Figure 1) [1]. PNA is highly stable chemically and, because of the unnatural backbone, highly resistant to enzymatic degradation, which makes it an excellent candidate for in vivo applications as an oligonucleotide analogue. The neutral pseudopeptide backbone eliminates electrostatic repulsion (a factor that negatively affects oligonucleotide binding) and PNA binds to DNA and RNA with excellent affinity. PNA binds to double helical DNA via two competing binding modes, triple helix (PNA : DNA, 1 : 1), and strand invasion, where PNA displaces one of the DNA strands, typically followed by a triplex formation (PNA : DNA, 2 : 1) [1]. PNA also forms exceptionally strong and sequence-specific Watson-Crick duplexes with single-stranded DNA and RNA [2]. Interestingly, the sequence specificity of duplexes involving PNA is substantially higher than that of unmodified nucleic acids. Because of these superior qualities, PNA has become a powerful tool in chemical biology and biotechnology [3–5]. The main applications of PNA are as hybridization probes and molecular diagnostics of high affinity and selectivity for single-stranded DNA and RNA. PNA also holds a promise of becoming a novel gene therapy agent for targeting specific RNA molecules [3, 4].

Although PNA binds single-stranded DNA and RNA with superior affinity and selectivity, there are other properties of PNA that can be further improved. Most importantly, in vivo applications of unmodified PNA are hindered by poor cellular uptake and endosomal entrapment [6]. Current methods to enhance the cellular uptake of PNA, such as conjugation with cell penetrating peptides (CPP) [7, 8], are complicated and require high PNA-peptide concentrations that may cause off-target binding and toxicity in vivo. Another problem is the limited sequence scope of double-stranded nucleic acids that can be recognized by PNA. While PNA can bind any sequence of single-stranded DNA and RNA with high affinity and selectivity, recognition of double helical DNA has been limited to polypurine tracts and binding to double helical RNA has been little explored. The present paper focuses on most recent developments in chemical modification of PNA to enhance cellular uptake and recognition of double helical nucleic acids. Several comprehensive reviews have recently discussed modification of PNA backbone [9, 10] and nucleobases [11] in a broader context.

## 2. Conjugation of PNA with Cationic Peptides to Improve the Cellular Uptake

Inefficient crossing of cellular membrane of mammalian cells by unmodified PNA has been a major problem for practical in vivo applications of PNA. Because of the neutral backbone, PNA does not associate with delivery vehicles based on cationic lipids. To use such standard oligonucleotide transfectants as Lipofectamine, PNA needs to be hybridized to complementary oligodeoxynucleotide (ODN) that aids the electrostatic complexation with the positively charged lipids [12]. Recently, a new approach to PNA delivery was developed by Wooley, Taylor and coworkers [13] who used cationic shell-cross-linked knedel-like nanoparticles (cSCKs) to deliver either PNA-ODN hybrid or PNA covalently attached to cSCKs nanoparticles through a biodegradable disulfide linkage. cSCKs nanoparticles have a hydrophobic core and a positively charged cross-linked shell. The latter is highly functionalizable and mediates the cellular delivery through, most likely, an endocytotic mechanism. An elegant extension of this technology is reported in this special issue by Taylor and coworkers [14].

Perhaps, the most popular approach to enhance cellular delivery has been conjugation of PNA with cell penetrating peptides that deliver the conjugate through the endocytosis pathway [7, 8]. However, the low ability of PNA-CPP conjugates to escape from endosomes has been the bottleneck of this approach. Various endosomolytic compounds have been explored; unfortunately, most are too toxic for in vivo applications [7]. Conjugates with arginine-rich peptides have shown promising activity in HeLa cells in the absence of endosomolytic agents [15]. However, even in the most promising cases large amount of conjugates remained in endosomes, leaving plenty of room for further improvement [15]. The relatively high concentrations of PNA-CCP, which are required for efficient delivery, may cause off-target binding and toxicity in vivo. Moreover, CPPs are relatively large peptides, which complicate the preparation and use of PNA-CPP conjugates. Recently, several groups have demonstrated that relatively simple cationic modifications in PNA can substantially improve their cellular uptake and produce effect similar to that of longer and more complex CPPs.

**Figure 2:**
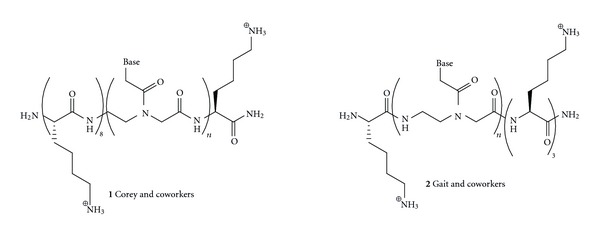
Conjugation of PNA with short oligolysines improves cellular uptake.

The groups of Corey [16, 17] and Gait [15, 18, 19] showed that conjugation of PNA with short oligolysine (Figure 2, **1** and **2**, resp.) enabled efficient delivery in fibroblast and various cancer cell lines (T47D, MCF-7, Huh7, and HeLa). As few as four lysine residues achieved similar efficiency as R6-Penetratin, a CPP previously optimized for cellular delivery of PNA [15]. Using short oligolysine instead of longer CPP significantly reduced the complexity and effort required for PNA use in cell culture. Lysine conjugates have also been used to deliver PNA in mice [20, 21]. Most recently, Gait and coworkers showed that introduction of a terminal Cys residue further increased the cellular uptake of Cys-Lys-PNA-Lys_3_ conjugate [22]. While some studies showed that conjugates built of the unnatural d-lysine were more effective [17], presumably due to higher biostability, other studies found little difference between the l and d series [22]. In a similar study, Fabbri et al. [23] demonstrated that PNA conjugated at the carboxyl terminus with octaarginine was efficiently taken up in human leukemic K562 cells and inhibited activity of the target microRNA-210.

Nielsen and coworkers have recently reported on conjugates of PNA with cationic ligands that showed improved cellular delivery and activity [24, 25]. In one study, addition of a lipid domain to the cationic peptides increased the activity of PNA conjugate by two orders of magnitude [24]. The lypophilic fatty acid contributed by promoting both endosomal uptake and endosomal escape of PNA. In another study, conjugation of PNA with polyethylenimine showed significantly higher antisense activity than PNA-octaarginine conjugates [25]. Polyethylenimine conjugates had lower toxicity than PNA-octaarginine conjugates. The polyethylenimine conjugate activity did not depend on the presence of lysosomolytic agents, which suggested that these conjugates are able to escape endosomes efficiently. These studies suggest that chemical approaches can be used to tailor cationic modifications that will improve cellular uptake and avoid the problem of endosomal entrapment.

Conjugation of PNA with a lipophilic triphenylphosphonium cation has been shown to increase the cellular delivery [26, 27]. In this special issue, Pandey, Patino and coworkers [28] report on cyclic and hairpin PNAs conjugated to the triphenylphosphonium cation via a disulfide linkage. The conjugates inhibit HIV replication by targeting the HIV-1 TAR RNA loop. Most recently, Shiraishi and Nielsen [29] reported on cellular uptake and antisense activity of PNA conjugated with cholesterol and cholic acid in HeLa pLuc705 cells. Although the conjugates alone were inactive, the delivery was dramatically improved by addition of Lipofectamine leading to nanomolar antisense activity.

As the numerous recent studies reviewed above suggest, design and optimization of CPP and other cationic ligands for cellular delivery of PNA is still a vigorous and important area of research. The focus has shifted to addressing endosomal escape, improving the end point activity and potential in vivo applications.

## 3. Cationic Backbone Modifications to Improve the Cellular Uptake of PNA

**Figure 3:**
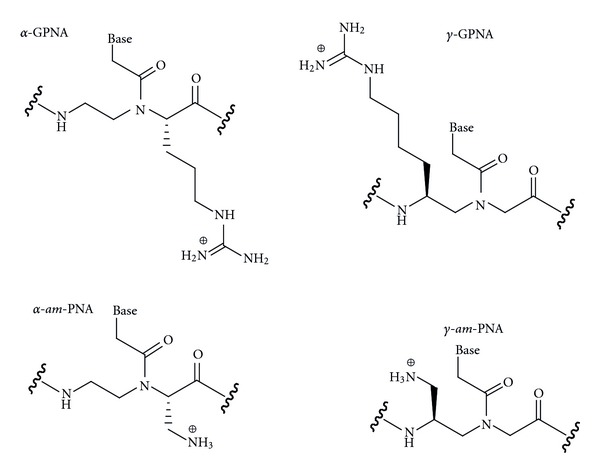
Cationic backbone modifications of PNA.

An alternative approach to conjugation of PNA has been direct modification of PNA's backbone. Several groups have explored cationic modifications of PNA [30–32]. Ly and coworkers introduced guanidine groups at *α*- [31] and *γ*-positions [32] of PNA's backbone by custom synthesis of monomers starting from diaminoethane and l or d arginine instead of glycine (Figure 3, l series shown). The *α*-guanidine-modified PNA (GPNA) derived from the unnatural d-arginine had higher affinity for complementary DNA [33] and RNA [34]; good sequence selectivity was maintained. GPNA was readily taken up by several cell lines (HCT116, human ES, and HeLa), which was attributed to the cationic guanidine modifications. GPNA was less toxic to cells than a PNA-polyarginine conjugate and induced potent antisense inhibition of E-cadherin in A549 cells [35]. Our laboratory recently studied the triple helix formation between double helical RNA and *α*-GPNA. We found that the *α*-guanidine modification decreased RNA binding affinity and sequence selectivity of *α*-GPNA compared to unmodified PNA [36].

The *γ*-guanidine-modified PNA had higher affinity for complementary DNA and RNA than *α*-guanidine-modified PNA, presumably due to favorable preorganization of the *γ*-modified backbone into a right-handed helix [32]. In contrast to *α*-modified PNA, Englund and Appella found that the *S*-isomer of *γ*-modified PNA (derived from the natural l-lysine) had higher affinity for complementary DNA than the *R*-isomer [30]. Most recently, Manicardi et al. [37] used both *α*- and *γ*-modified GPNA 15-mers to inhibit microRNA-210 in K562 cells. Both isomers showed promising though not complete inhibition with the PNAs having eight consecutive *γ*-modification at the carboxyl terminus performing slightly better than other modification patterns [37].

Mitra and Ganesh reported similar results on DNA binding and cellular uptake of *α*- and *γ*-aminomethylene PNA (*am*-PNA, Figure 3) [38, 39]. The aminomethylene modification increased PNA binding to DNA, with *γ*-(*S*)*am*-PNA being significantly better than *α*-(*R*)*am*-PNA, which, in turn, was better than *α*-(*S*)*am*-PNA [39]. The cellular uptake was enhanced by these modifications in roughly the same order, with *γ*-(*S*)*am*-PNA giving the most promising results.

## 4. PNA Modifications to Expand the Recognition of Double-stranded Nucleic Acids

**Figure 4:**
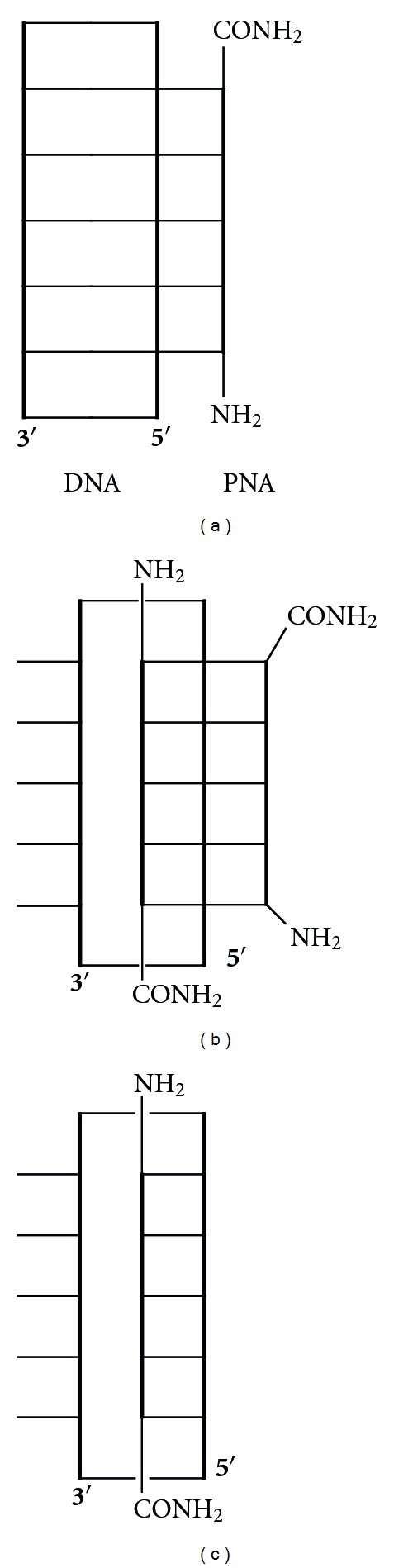
Binding modes for recognition of double-stranded DNA: triple helix (a), strand-displacement triplex (b), and strand-displacement duplex (c).

**Figure 5:**
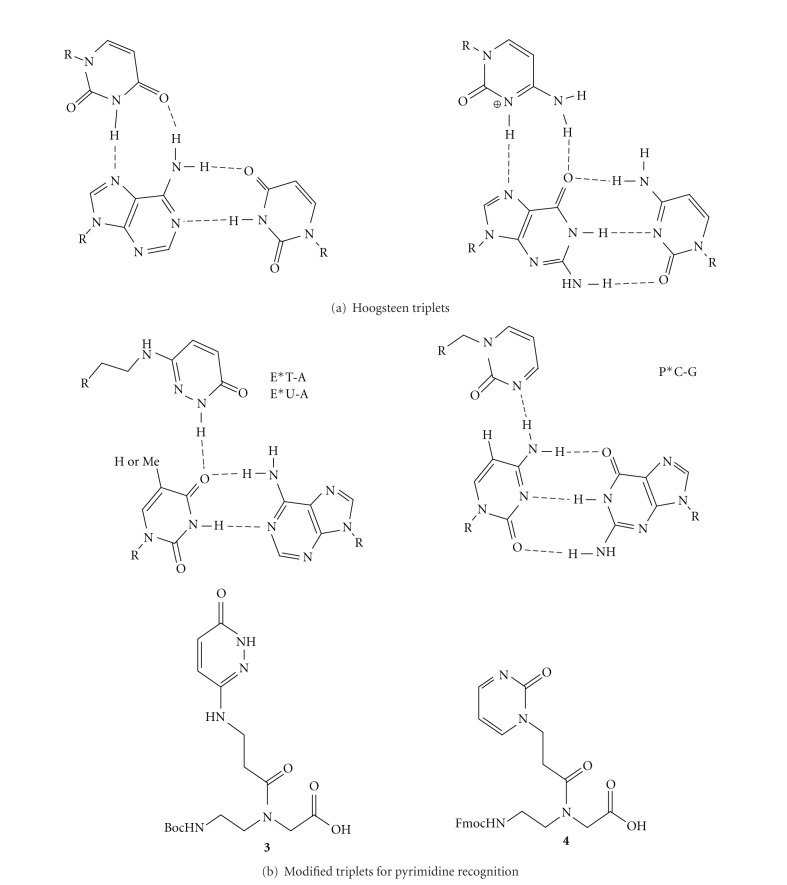
Recognition of purines and pyrimidines using Hoogsteen (a) and modified (b) triplets, respectively.

Recognition of single-stranded DNA and RNA following the Watson-Crick base pairing rules is relatively straightforward. Recognition of double-stranded nucleic acids is substantially more challenging because the Watson-Crick faces of nucleobases are already engaged in hydrogen bonding. PNA, as well as other oligonucleotide analogues, can recognize double-stranded nucleic acids by forming either a parallel triple helix (Figure 4(a), the amino end of PNA aligned with the 5′ end of DNA) or a strand-invasion complex, where PNA displaces one of the DNA strands. The strand-invasion is typically a competing mode for triplex (PNA : DNA, 1 : 1) and usually results in a strand-displacement triplex (PNA : DNA, 2 : 1). The PNA strand that is replacing the DNA strand aligns antiparallel with the DNA strand (Figure 4(b), the carboxyl end of PNA aligned with the 5′ end of DNA). Both binding modes are limited to nucleic acid duplexes featuring so-called polypurine tracts where one strand is built of purines, while the other strand consists of pyrimidines. This is because the standard Hoogsteen triplets (U*A-U and C+*G-C) recognize only purine bases (Figure 5(a)).

The strand-displacement triplex approach (Figure 4(b)) typically uses PNA clamps that have the two PNA strands connected by a short linker, which enhances the binding affinity and favors strand invasion. To expand the repertoire of sequences that can be recognized by the triplex forming part of PNA, Dahl and Nielsen designed 3-oxo-2,3-dihydropyridazine nucleobase (**E**, Figure 5(b)) to recognize thymidine in T-A base pairs of DNA [40]. This modification substantially increased the thermal stability of a PNA clamp targeting 10-nucleotide long DNA stretch that had two thymidines interrupting the purine-rich strand [40]. Despite the promising preliminary results, this modification has not been widely applied either in strand-displacement triplex or in triple helical approaches.

**Figure 6:**
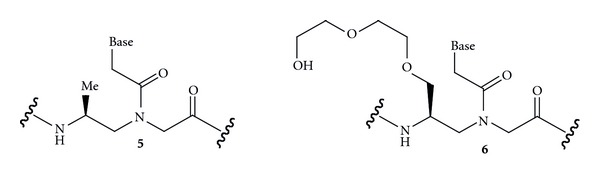
*γ*-Alkyl PNA for recognition of double-stranded DNA via strand-displacement duplex.

A PNA : DNA 1 : 1 strand-displacement duplex (Figure 4(c)) would be a highly desired binding mode because, in principle, any sequence of DNA could be recognized without the need for the presence of a purine-rich strand. However, this recognition mode is complicated by the fact that duplex forming PNA does not have enough thermodynamic advantage to displace a DNA stand from a duplex. Ly and coworkers recently showed that *γ*-methylation (Figure 6, **5**) preorganized PNA into right-handed helix and enhanced its ability to form strand-displacement complex with mixed sequence DNA [41]. The properties of invading *γ*-modified PNAs were further improved by incorporation of G-clamp nucleobases [42] and replacement of the methyl group with MiniPEG (Figure 6, **6**) [43]. The latter modification was critical to optimize water solubility and minimize PNA aggregation and enabled PNA built of monomers **6** invade essentially any sequence of double-stranded DNA in a highly sequence-specific manner [43].

The triple helical recognition of double-stranded DNA using PNA has received less attention than the strand-displacement approaches. However, in a recent and comprehensive study Nielsen and coworkers showed that this is a promising and perhaps underutilized approach [44]. Compared to DNA, molecular recognition of double-stranded RNA has been even less studied. This is perhaps because for a long time RNA was believed to be only a passive messenger in the transfer of genetic information from DNA to proteins. However, since the discovery that RNA can catalyze chemical reactions, the number and variety of noncoding RNAs and the important roles they play in biology have been growing steadily. While less than 2% of DNA encodes for functional proteins, almost 70% is transcribed into RNA. Today, the functional importance of most RNA transcripts is still unknown and it is fairly safe to predict that we will discover many more regulatory RNAs in the near future. The ability to selectively recognize, detect, and inhibit the function of such RNAs will be highly useful for both fundamental biology and practical applications in biotechnology and medicine.

Recently, our laboratory started studies on triple helical recognition of double-stranded RNA using PNA [45, 46]. Before this effort, triple helices between RNA and PNA were virtually unknown; there was only one study by Toulme and coworkers that suggested that PNA may not be forming stable triple helix with RNA [47]. In contrast, we found that PNA formed a highly stable and sequence-selective triple helix with double-stranded RNA [45]. Interestingly, the RNA-PNA triplexes were at least an order of magnitude more stable than the DNA-PNA triplexes suggesting that PNA may be a significantly better ligand for the deep and narrow major groove of RNA than for the wider major groove of DNA [45]. To expand the sequence scope of RNA that can be recognized, we adopted monomer **E** for recognition of uridine in U-A base pair and designed a novel monomer, 2-pyrimidone **P** for recognition of cytidine in C-G base pair (Figure 5(b), **3** and **4**, resp.) [46]. Our design of **P** was inspired by the work of Leumann and coworkers [48, 49] who used 4-methyl-2-pyrimidone as an oligonucleotide modification for triple helical recognition of cytidine in C-G base pairs of DNA. Heterocycle **P** had not been used in PNA before our study. Incorporation of **E** and **P** in short PNA sequences allowed recognition of nine-nucleotide long polypurine tracts of double helical RNA containing single pyrimidine inversion. The selectivity was good and affinity matched that of the standard Hoogsteen triple helices (Figure 5(a)) [46]. Our results also showed that the extended linkers connecting **E** and **P** heterocycles to the PNA backbone were important design elements that optimized the binding affinity [46].

## 5. Conclusions

Since invention of PNA, synthetic chemists have been extensively modifying its structure [9–11]. Most of the work on backbone modifications of PNA has attempted, with mixed success, to improve the affinity and selectivity of Watson-Crick recognition of DNA and RNA. Reviewed herein are selected recent studies focused on improving cellular uptake of PNA and developing novel modes of binding, such as strand-invasion of mixed sequence double-stranded DNA and triple helical recognition of RNA. The preliminary results are very encouraging, and it is likely that more improvements and new discoveries will be made in the near future.
